# Supplementary material for: The ABA-LANCL1/2 Hormone-Receptors System Protects H9c2 Cardiomyocytes from Hypoxia-Induced Mitochondrial Injury via an AMPK- and NO-Mediated Mechanism
Source: Cells. 2022 Sep 15;11(18):2888. doi: 10.3390/cells11182888 (PMC9496903; doi:10.3390/cells11182888)
Supplement: Supplementary file 1 [file cells-11-02888-s001.zip › cells-1897430-supplementary.pdf]

SUPPLEMENTARY DATA

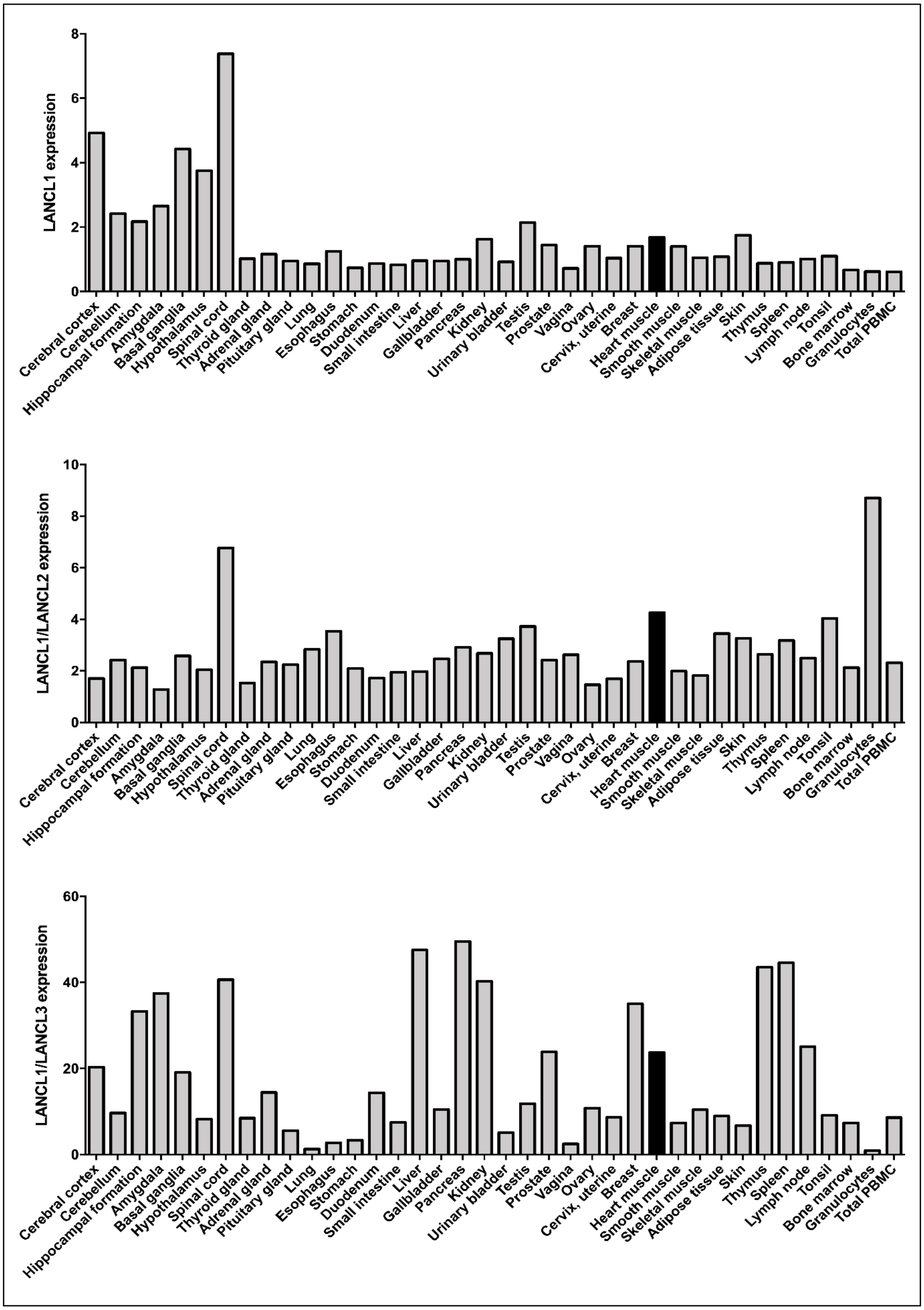

Figure S1. Tissue expression levels of LANCL proteins could mediate a tissue-specific function.

Table S1. Primer sequences used to amplify rat target genes.

Rat genes	Accession N.	Forward Primer 5′-3′	Reverse Primer 5′-3′
Lancl1	NM_053723	TCTTGCTCCTCATCCTGCTCATC	CACTGTACTCGCCGAAGGTCTC
Lancl2	NM_001014187	GGTGCCACGGTGCTCCAG	CCTCGCTGCCAAATCACATCAC
Prkaa2	NM_019142	AGAAGCAGAAGCACGACGG	GAAGGTGCCGACGCCC
Ppargc1a	NM_031347	GCACACATCGCAATTCTCCC	CTCTGCGGTATTCGTCCCTC
Sirt1	NM_001372090	CAGTGTCATGGTTCCTTTGC	CACCGAGGAACTACCTGAT
Nampt	NM_177928	TCGGTTCTGGTGGAGGTTTGCTAC	TCCCTGCTGGCGTCCTATGTAAAG
Hprt1	NM_012583	TTGGTCAAGCAGTACAGCCC	TGGCCTGTATCCAACACTTCG
Tbc1d1	XM_032917081	AGTCAGGACCCGAGCTACTT	CTGCCGGATGGAGCTAAT
Nos1	NM_052799	AGAGGAGGACGCTGGTGTAT	GA
Nos2	NM_012611	GGAGAAAACCCCAGGTGCTA	AAGGCGGTTGGTCACTTCAT
			TGAGGAACTGGGGGAAACCA
Nos3	NM_021838	AGGCCTTGGTATTGGTGGTG	TAGGGGCCCGACATTTCCAT
Gch1	NM_024356	GCGTCGGGGTAGTGATTGAA	AGCATGGTGCTAGTGACAGT
Arg2	U90887	AAAAGGCAGAGGCCAATCCA	TCCCCCTACAACAGGGGTTC
Slc7a2	NM_001134686	TCCCTCTGCGCCTTATCAAC	TTTGAAAAGCAACCCATCCTCC
Nadk2	NM_001044252	AAGAGCGCACGATGAAAGGT	GCTACAGCCCAACAATAAGGC

Table S2. Primary and secondary antibodies used for Western blot.

Primary Antibody	Host	Concentrations	Manufacturer
Anti-LANCL1	Rabbit	1:250	Novus Biologicals
Anti-LANCL2	Mouse	1:1000	Reference [19]
Anti-AMPK tot	Rabbit	1:1000	Cell Signaling Technology, Danvers, MA
Anti-pAMPK P-Thr172	Rabbit	1:1000	Cell Signaling Technology, Danvers, MA
Anti-vinculin	Rabbit	1:1000	Cell Signaling Technology, Danvers, MA
Anti-Akt tot	Rabbit	1:1000	Cell Signaling Technology, Danvers, MA
Anti-pAkt P-Ser473	Rabbit	1:1000	Santa Cruz Biotechnology Inc., California
Anti-eNOS tot	rabbit	1:1000	Cell Signaling Technology, Danvers, MA
Anti-peNOS P-Ser1177	rabbit	1:1000	Cell Signaling Technology, Danvers, MA
Anti-α tubulin	rabbit	1:200	Cell Signaling Technology, Danvers, MA

Secondary Antibody	Concentrations	Manufacturer
Anti-Mouse	1:2000	Santa Cruz Biotechnology Inc., California
Anti-Rabbit	1:1000	Santa Cruz Biotechnology Inc., California
